# Connectome-derived diffusion characteristics of the fornix in Alzheimer's disease

**DOI:** 10.1016/j.nicl.2018.04.029

**Published:** 2018-04-27

**Authors:** Rodrigo D. Perea, Jennifer S. Rabin, Megan G. Fujiyoshi, Taylor E. Neal, Emily E. Smith, Koene R.A. Van Dijk, Trey Hedden

**Affiliations:** a Athinoula A. Martinos Center for Biomedical Imaging, Dept. of Radiology, Massachusetts General Hospital, Charlestown, MA, United States; b Dept. of Radiology, Harvard Medical School, Boston, MA, United States; c Dept. of Psychiatry, Massachusetts General Hospital, Boston, MA, United States; d Dept. of Psychiatry, Harvard Medical School, Boston, MA, United States; e Dept. of Psychiatry, University of Texas Southwestern Medical Center, Dallas, TX, United States

**Keywords:** MRI, Aging, Hippocampus, White matter, Connectivity

## Abstract

The fornix bundle is a major white matter pathway of the hippocampus. While volume of the hippocampus has been a primary imaging biomarker of Alzheimer's disease progression, recent research has suggested that the volume and microstructural characteristics of the fornix bundle connecting the hippocampus could add relevant information for diagnosing and staging Alzheimer's disease. Using a robust fornix bundle isolation technique in native diffusion space, this study investigated whether diffusion measurements of the fornix differed between normal older adults and Alzheimer's disease patients when controlling for volume measurements. Data were collected using high gradient multi-shell diffusion-weighted MRI from a Siemens CONNECTOM scanner in 23 Alzheimer's disease and 23 age- and sex-matched control older adults (age range = 53–92). These data were used to reconstruct a continuous fornix bundle in every participant's native diffusion space, from which tract-derived volumetric and diffusion metrics were extracted and compared between groups. Diffusion metrics included those from a tensor model and from a generalized q-sampling imaging model. Results showed no significant differences in tract-derived fornix volumes but did show altered diffusion metrics within tissue classified as the fornix in the Alzheimer's disease group. Comparisons to a manual tracing method indicated the same pattern of results and high correlations between the methods. These results suggest that in Alzheimer's disease, diffusion characteristics may provide more sensitive measures of fornix degeneration than do volume measures and may be a potential early marker for loss of medial temporal lobe connectivity.

## Highlights

• An enhanced method for measurement of continuous fornix bundles is described.
• Diffusion characteristics of the fornix were degraded in Alzheimer's disease.
• Alzheimer's disease primarily affected the crus and body of the fornix.
• Diffusion differences were observed controlling for fornix volume differences.

## 1 Introduction

Alzheimer's disease is a progressive neurodegenerative disorder characterized by a profound loss of episodic memory accompanied by the presence of amyloid-β plaques, neurofibrillary tangles, and atrophy of medial temporal lobe memory circuit structures (Hyman et al., 1990; McKhann et al., 2011; Hyman et al., 2012; Aggleton et al., 2016). While amyloid-β plaques are necessary for pathological confirmation of Alzheimer's disease, the combination of amyloid burden with medial temporal lobe neurodegeneration predicts faster cognitive decline and clinical progression (Knopman et al., 2012; Rowe et al., 2013; Mormino et al., 2014). This suggests that more complete characterization of the connectivity and function of medial temporal lobe structures is important to the development of biomarkers for diagnosing and understanding the progression of Alzheimer's disease (Bozzali et al., 2016; Jagust, 2016) distinct from age-related alterations (Madden et al., 2012; Fjell et al., 2017).

Although gray matter volume of the hippocampus has been an important biomarker of medial temporal lobe neurodegeneration (Scheltens et al., 1992; Bobinski et al., 2000; Gosche et al., 2002; Jack et al., 2002), alterations of hippocampal white matter pathways are often observed in Alzheimer's disease (Hopper and Vogel, 1976; Ringman et al., 2007; Zhang et al., 2007; Sexton et al., 2011; Nowrangi and Rosenberg, 2015). The fornix bundle forms a direct pathway connecting the hippocampus to the mammillary bodies, the thalamic nuclei, and the prefrontal cortex (Papez, 1937; Kantarci, 2014; Pessoa and Hof, 2015; Aggleton et al., 2016). Lesions to the fornix produce spatial and scene memory deficits in animal models (Gaffan, 1994; Neave et al., 1997; Aggleton and Brown, 1999) and verbal and nonverbal amnesia in humans (Yamamoto et al., 1990; D'Esposito et al., 1995; McMackin et al., 1995; Aggleton and Brown, 1999). More subtle but still disruptive alterations of white matter tracts including the fornix (Braak and Braak, 1996) typically occur during Alzheimer's disease, including axonal degeneration and demyelination (Hopper and Vogel, 1976; Brun and Englund, 1986; Bartzokis, 2004; Kanaan et al., 2013; Amlien and Fjell, 2014; Zhang et al., 2014). Several studies have reported decreased volumetric measurements of the fornix in Alzheimer's disease and in cognitively normal individuals who later experience cognitive decline (Copenhaver et al., 2006; Fletcher et al., 2013; Amaral et al., 2016). However, there is inconsistent evidence for disruption of the fornix in Alzheimer's disease from diffusion-weighted imaging, with reviews noting differences in findings across studies that may be ascribed to constitution of diagnostic groups, methodologies for identifying the fornix, likelihood of contamination from CSF, or to diffusion characteristics tested (Acosta-Cabronero and Nestor, 2014; Oishi and Lyketsos, 2014; Nowrangi and Rosenberg, 2015). Diffusion-weighted MRI provides information regarding microstructural alterations of the white matter (Le Bihan et al., 1986; Basser et al., 1994) by using the directional diffusion of water to identify constrained tissue such as the dense myelinated fibers of the fornix bundle (Budde et al., 2007; Concha, 2014; Walhovd et al., 2014; Seehaus et al., 2015; Chang et al., 2017). Several studies have found relatively impaired diffusion characteristics in the fornix in Alzheimer's disease patients by measuring regions of interest within the fornix bundle (Mielke et al., 2009; Huang et al., 2012; Nowrangi et al., 2013), or applying template-based methods (van Bruggen et al., 2012; Douaud et al., 2013; Jin et al., 2015; Brown et al., 2017). Some evidence further suggests that microstructural alterations in the fornix bundle may be more sensitive than macroscopic hippocampal atrophy to Alzheimer's disease (Bozoki et al., 2012; van Bruggen et al., 2012; Fletcher et al., 2013; Zhuang et al., 2013).

The inconsistent pattern of findings regarding diffusion in the fornix bundle may be due to its intrinsic morphological properties that impact the analysis and interpretation of diffusion-weighted images. These properties include: 1) that the fornix bundle is a highly curved white matter structure, making it difficult to apply tractography algorithms that rely on angular thresholds; 2) that diverging fiber populations in the medial region of the fornix bundle known as the hippocampal commissure may complicate estimation of directional diffusion, and 3) that its adjacency to cerebrospinal fluid (CSF) makes it highly susceptible to partial-volume effects. Measurement problems associated with these properties become exacerbated with lower fiber density or decreased diffusion signal, as occurs during aging and Alzheimer's disease (Bartzokis, 2004; Acosta-Cabronero et al., 2010; Bartzokis, 2011; Salat, 2011; Amlien and Fjell, 2014; Sexton et al., 2014). Additionally, the challenges posed by these properties increase at lower diffusion gradient strengths, indicating potential for improvement from hardware-enhanced collection of diffusion-weighted images. To address these challenges, we performed streamline tractography using diffusion-weighted images acquired from the first-of-its-kind MGH-USC CONNECTOM MRI scanner (Setsompop et al., 2013). By applying high b-value gradient directions (Fan et al., 2014), a multi-shell/multi-fiber reconstruction model (Yeh et al., 2010), and semi-automatic processing protocols for tractography in native diffusion space, we were able to reconstruct a continuous fornix bundle and estimate diffusion characteristics along its trajectory for individual participants.

The primary question of this study was: How do volumetric decreases of the fornix bundle associated with Alzheimer's disease relate to the measurement of diffusion characteristics in the fornix? We sought to answer this question by comparing Alzheimer's disease patients relative to age- and sex-matched older adults using the high-gradient diffusion measurement afforded by the CONNECTOM scanner and by applying multiple diffusion models. We considered three possible relationships: 1) despite diminished volume of the fornix in Alzheimer's disease, the remaining fibers could nonetheless have intact diffusion characteristics indicative of intact microstructure (e.g. myelination, axonal density, etc.), 2) diffusion characteristics of the fornix could be altered even in Alzheimer's disease participants with intact volume, or 3) both volume and diffusion characteristics could be altered in participants with Alzheimer's disease. Prior studies have found both decreased volume and impaired diffusion characteristics consistent with the third possibility. However, reduced volume likely induces partial volume effects from CSF contamination that would manifest as impaired diffusion values. Here, we attempted to control for volume and examined multiple diffusion measurements, including non-tensor metrics, when describing the diffusion characteristics of the fornix. We applied a semi-automated reconstruction method that could potentially be applied to large-scale data sets, and validated the quantitative metrics derived from this reconstruction against manual tracings of the fornix bundle.

## 2 Materials and methods

### 2.1 Participants

Participants were 23 possible or probable Alzheimer's disease patients and 23 age- and sex-matched healthy controls. Participants were recruited from existing cohorts and clinics, including from the Harvard Aging Brain Study (Dagley et al., 2017), the Massachusetts Alzheimer's Disease Research Center, and the Memory Disorders Unit clinic at Massachusetts General Hospital. Alzheimer's disease patients were included if they had a global Clinical Dementia Rating (CDR) ≥ 1 (M = 1.43, SD = 0.59), a clinical diagnosis of possible or probable Alzheimer's disease (5 presented with mixed cognitive symptoms judged secondary to Alzheimer's disease), and a score ≤ 22 on the Mini-Mental State Examination (MMSE; Folstein et al., 1975). Normal controls matched in age and sex to the Alzheimer's disease patients were selected from a larger pool of 47 control participants, and had a global CDR = 0, MMSE ≥ 26. For one matched pair, matching on age was prioritized over matching on sex. Amyloid imaging using positron emission tomography with Pittsburgh Compound B was available prior to enrollment for some control participants; control participants classified as amyloid positive using standardized methods (Hedden et al., 2016) were not contacted for enrollment and would therefore have been excluded from the larger pool of control participants. The study was conducted in accordance with Institutional Review Board approval at Massachusetts General Hospital. All participants provided informed written consent or assent with surrogate consent, and were screened for magnetic resonance contraindications. Table 1 summarizes participant characteristics.

**Table 1:** Demographics, diffusion head motion during diffusion MRI acquisition, and Mini-Mental State Examination (MMSE) for the Alzheimer's disease and normal control groups. SD = standard deviation.

	Alzheimer's disease (n = 23)	Normal controls (n = 23)	p-Value
Age range	53–91	53–92	–
Mean age (SD)	73.74 (10.3)	74 (10.7)	0.93
Sex, M:F	9:14	10:13	0.76
Mean head motion (mm)	2.56 (1.5)	1.76 (1.3)	0.053
Mean MMSE (SD)	17 (5.5)	29.3 (0.8)	<0.001

### 2.2 Imaging data and processing

#### 2.2.1 Imaging data acquisition

Data were acquired on the Siemens 3-Telsa CONNECTOM scanner, a custom installation based on the Skyra platform (see Setsompop et al., 2013) using a custom 64-channel phased array head coil (Keil et al., 2013). Briefly, we acquired a T1-weighted 1-mm isotropic Multi-Echo Magnetization Prepared Rapid Acquisition Gradient Echo (MEMPRAGE) image with a repetition time (TR) of 2530 ms and echo times (TE) of 1.61, 3.47, 5.34, and 7.19 ms. A T2-weighted 1-mm isotropic spin-echo image (TR = 3200 ms, TE = 560 ms) was acquired. Diffusion-weighted images were acquired with 1.8 mm^3^ resolution at a gradient strength of 243 mT/m in 4 runs using two b-values: 3 runs at 7500 s/mm^2^ (TR = 3300 ms, TE = 54 ms, 60 unique gradient directions per run for 180 total directions) and 1 run at 2500 s/mm^2^ (TR = 3200 ms, TE = 49 ms, 60 directions). Total diffusion acquisition time was 18.97 min across the four runs. This high diffusion gradient acquisition affords increased diffusion-weighted sensitivity with sharper spin distribution functions (SDFs) and increased signal-to-noise ratio compared with conventional acquisitions (Fan et al., 2014). In each run, four initial b0 gradient images (b = 0 s/mm^2^) were acquired (with the first discarded) and repeated at every 13th image for use in motion correction. Initial pre-processing included non-linearity gradient correction, head motion correction, and eddy current correction (Fan et al., 2016; Andersson and Sotiropoulos, 2016). Head motion during diffusion imaging was calculated as the absolute motion based on the translation parameter for each dimension (x, y, z) extracted from the FSL tool eddy (Andersson and Sotiropoulos, 2016). Between modality registration of the T1, T2, and diffusion images was accomplished using the spm_coreg function in SPM12 (Wellcome Trust Centre for Neuroimaging, London, UK).

#### 2.2.2 Imaging reconstruction

After initial pre-processing and to take advantage of the multi-shell acquisition, we used the generalized q-sampling imaging (GQI) model to reconstruct whole brain spin density functions (SDFs) with a sampling length ratio of 1.25. In contrast to orientation distribution functions (ODFs) that represent the probability distribution of diffusion displacement, SDFs represent a quantitative distribution of spins undergoing diffusion (Yeh et al., 2010). GQI's accuracy to resolve major and minor fibers is comparable with Q-ball imaging in the shell sampling scheme (Tuch, 2004) and diffusion spectrum imaging (DSI) in the grid sampling scheme (Wedeen et al., 2005).

#### 2.2.3 Tractography regions of interest (ROIs) and avoidance (ROAs)

**Fig. 1:**
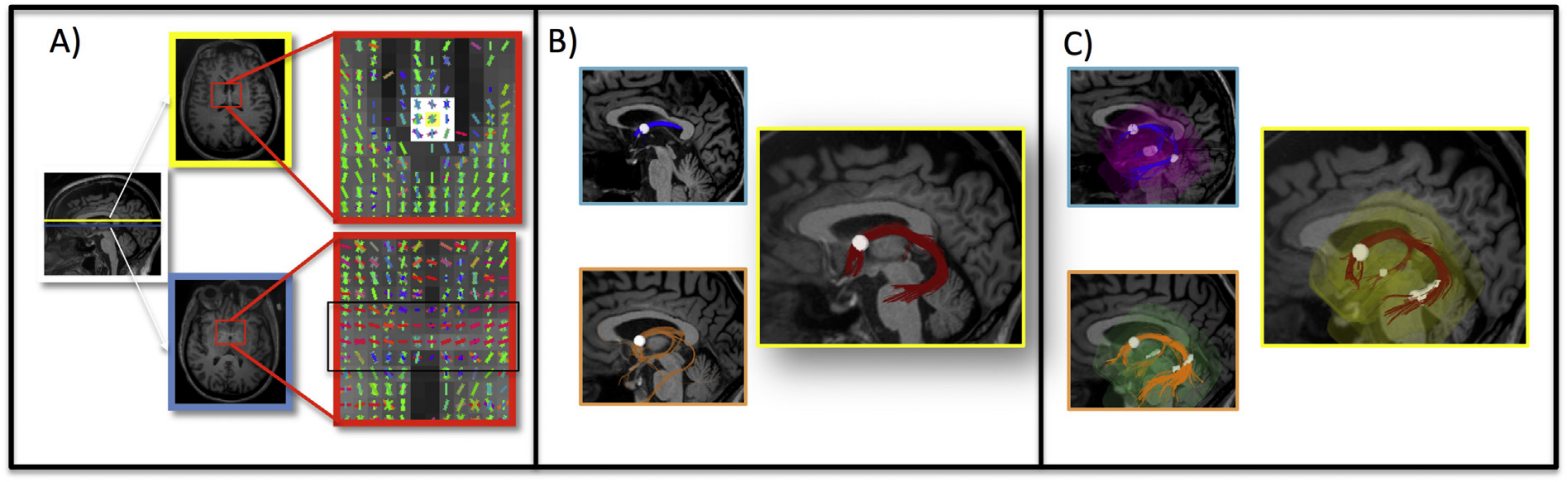
Method for fornix bundle reconstruction. For every participant, A) after localizing the anterior commissure in the horizontal slice (blue box), the plane was moved three slices superiorly (yellow box) and a spherical region of interest (ROI, white region inside red box) was created to perform tractography. B) The normal control with the most canonical fornix reconstruction from this spherical ROI (yellow box; two other participants are shown for reference in the blue and orange boxes) was selected, morphologically dilated nine iterations, co-registered to every participant's b0 image, and hollowed to create a subject-specific region of avoidance (ROA). C) We then applied deterministic tractography using the spherical ROI and fimbriae ROIs from the FreeSurfer 6.0 parcellation as seeds inside the subject-specific ROA (three representative participants are shown). (For interpretation of the references to color in this figure legend, the reader is referred to the web version of this article.)

To provide consistent anatomical landmarks across participants, ROIs were placed in the body of the fornix above and behind the anterior commissure and at the left and right fimbria. ROIs are defined as all voxels in a volumetric mask where the tractography algorithm (see Section 2.2.4) initializes. ROAs are hollow surfaces (usually surrounding ROIs) to filter out streamlines not consistent with the morphology of the bundle of interest. Automatically generated ROIs for the fimbriae were estimated using hippocampal subfield segmentations (Iglesias et al., 2015) from FreeSurfer 6.0 (Fischl et al., 2002) and warped into native diffusion space by co-registration to the initial b0 diffusion image using bbregister (Greve and Fischl, 2009). Two Alzheimer's disease patients failed to have any voxels assigned to the left fimbria ROI and were excluded from all analyses of the left fornix. Five voxels or less were assigned to a fimbria ROI for 10 (left) and 12 (right) Alzheimer's disease patients and for 1 (left) and 7 (right) normal controls; these were dilated by a 3 × 3 × 3 kernel using fslmaths, followed by removal of any resulting voxels that were outside the boundaries of the full hippocampus segmentation. To create a spherical ROI at the anterior fornix body, on every participant's generalized fractional anisotropy (GFA) image overlaid with the color-coded SDFs, we localized the anterior commissure in the horizontal slice and confirmed its left-right direction using the principal SDFs (Fig. 1A, blue box). We moved three slices above the anterior commissure (~5.4 mm superior) and drew a spherical ROI (three voxel diameter) by tracing the anterior-to-posterior SDFs (Fig. 1A, yellow box). Streamlines were visually inspected to confirm adequate reconstruction. To generate ROAs, we selected the reconstructed fornix of the normal control with the most canonical reconstruction (Fig. 1B, yellow box) as a template. Using DSI Studio's spatial filtering (Yeh et al., 2013), this template was dilated by nine iterations to create a region large enough to surround the fornix bundle in all participants. The canonical dilated fornix template was co-registered to every participant's native diffusion space, and hollowed by zeroing voxels from the same co-registered canonical region eroded by a 3 × 3 × 3 kernel using fslmaths (Fig. 1C). This creates a subject-specific hollow region large enough to surround the fornix bundle in all our participants and permit adequate streamline reconstruction, while filtering out false positive streamlines that extend beyond the fornix morphology. The ROI fornix bundle template and the ROA are available as Supplementary material.

#### 2.2.4 Reconstruction of a continuous fornix bundle (tractography)

**Fig. 2:**
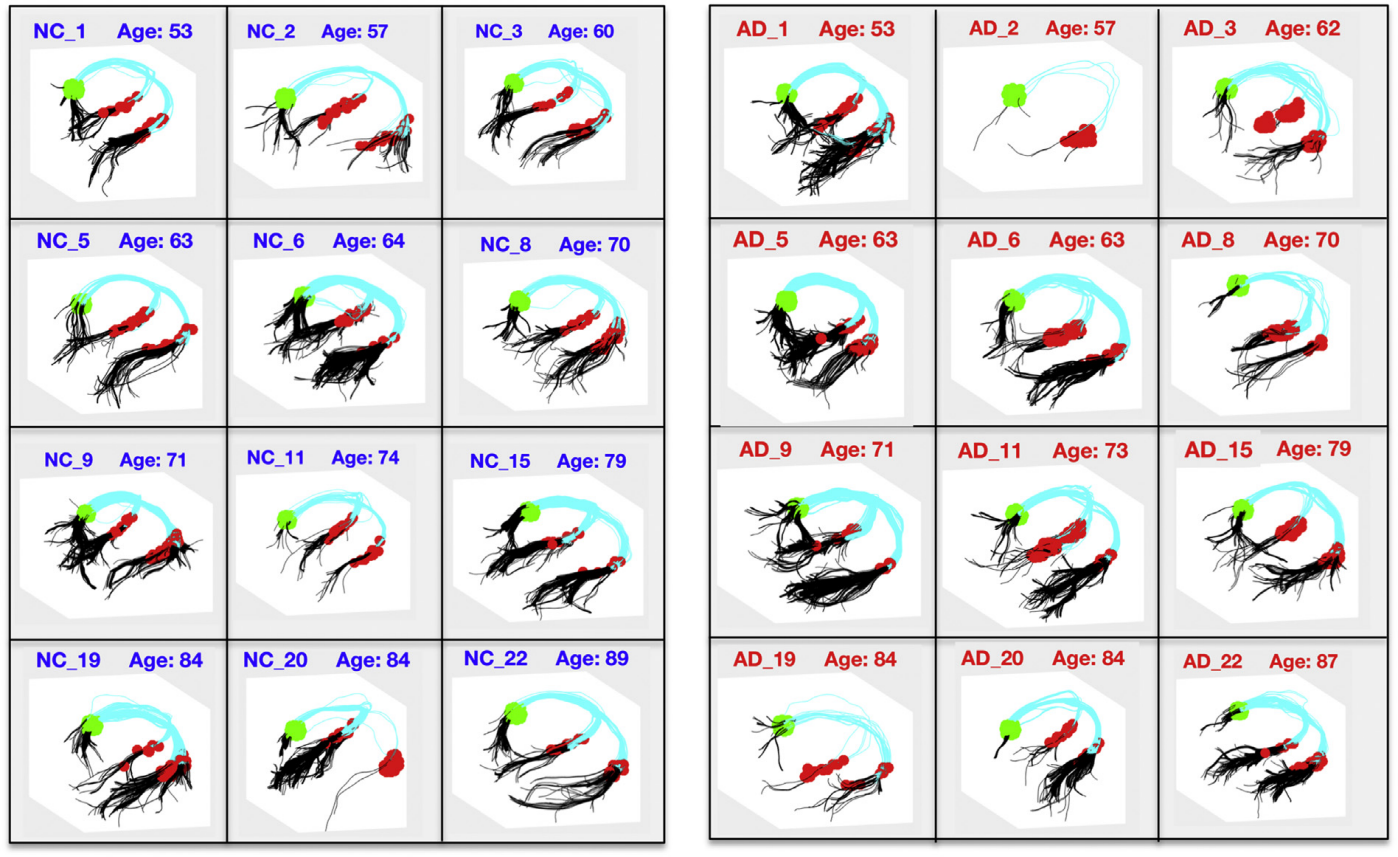
Representative examples of continuous fornix bundle reconstruction in 12 normal controls (NC_#) and age-matched Alzheimer's disease patients (AD_#). Reconstructions were performed in every participant's native diffusion space. Green denotes the spherical ROI, red denotes the automatically generated fimbriae ROIs, cyan and black denote the reconstructed fornix bundles with black showing the portion of each streamline extending beyond the ROIs that was trimmed in some analyses. (Left hemisphere is on the left.) (For interpretation of the references to color in this figure legend, the reader is referred to the web version of this article.)

To provide reconstruction of a continuous fornix bundle, using DSI Studio we applied streamline fiber tracking based on the Euler method for solving ordinary differential equations (Basser et al., 2000). We set the angular threshold to 40 degrees, 1-mm step size and set the termination index to 0.02 of the normalized principal quantitative anisotropy (NQA0), a normalized metric that quantifies the diffusion spin population along the principal diffusion direction (Yeh et al., 2013). After placing the corresponding ROA, we used the left and right fimbria ROIs separately as seed regions (Fig. 2, red color), allowing tractography to begin here while filtering in only streamlines that reached the spherical ROI generated from randomly assigning 10,000 seeds on each ROI (Fig. 2, green color). Example reconstructions of these continuous bundles are shown in Fig. 2. The algorithm failed to reconstruct a continuous fornix bundle in the left hemisphere for three Alzheimer's disease participants (one failed to reconstruct continuous streamlines while two had missing left fimbriae ROIs). To maintain matching between the groups, we excluded the corresponding age- and sex-matched normal controls when performing statistical tests. Streamlines were examined for consistency with the curved morphology of the fornix bundle. Streamlines not fully conforming to this morphology were taken as candidate false positive streamlines. The average count of candidate false positive streamlines relative to the total number of streamlines in both hemispheres was 2.5/673 (0.3%) in the normal controls, with 65% of controls having at least one such candidate false positive, and a maximum of 6 candidate false positive streamlines for any control participant. The average count was 2.7/682 (0.4%) in the Alzheimer's disease patients, with 43% of Alzheimer's patients having at least one candidate false positive, and a maximum of 5 false positive streamlines for any Alzheimer's patient.

#### 2.2.5 Most robust streamline isolation and localized fornix bundle comparison protocol

**Fig. 3:**
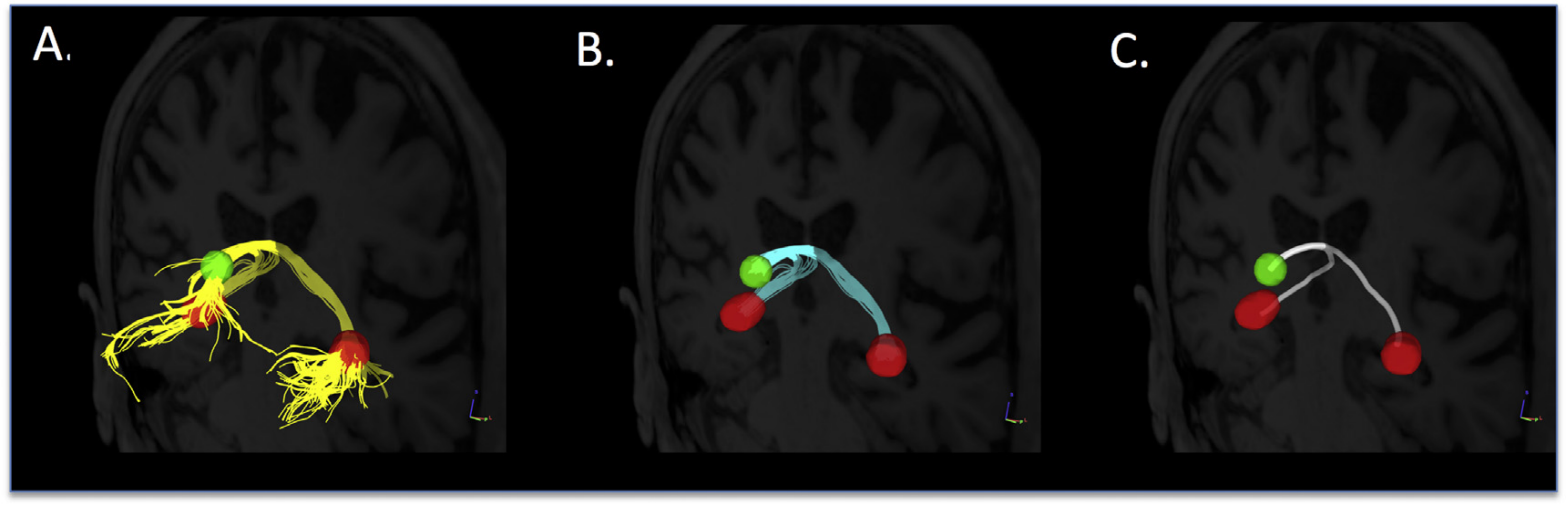
Demonstration of streamline tractography and reconstruction of continuous fornix bundles. In every participant's native diffusion space, A) streamline tractography (yellow streamlines) was performed using the spherical (green) and fimbriae (red) regions of interest as separate seeds and limited by a subject-specific region of avoidance (not shown). B) To enable group comparisons guided by anatomically-based landmarks, the portion of each streamline that extended beyond the ROIs was trimmed, keeping only that portion between the ROIs (cyan). D) Finally, the single streamline with the highest average fractional anisotropy (white streamline) was selected for each participant and used to extract quantitative metrics for statistical analyses. (For interpretation of the references to color in this figure legend, the reader is referred to the web version of this article.)

Applying the above methods (Section 2.2.4) resulted in reconstruction of a continuous fornix bundle (Fig. 3A, yellow). We trimmed the portion of each streamline extending beyond the ROIs (Fig. 2C, black and Fig. 3A, yellow). The remaining portion (Figs. 2B and 3B, cyan) identified a continuous fornix bundle anchored at the same anatomical landmarks in each participant. Next, we performed B-spline interpolation of the coordinates of each participant's trimmed streamlines to forty data points (based on the average number of voxels within each fornix streamline), following methods similar to previous reports (Colby et al., 2012). Finally, to enable statistical comparisons across groups while minimizing partial volume effect susceptibilities, we identified the streamline with the highest averaged fractional anisotropy (aka most robust streamline) in each hemisphere in each participant, from which diffusion metrics were extracted (similar to Davis et al., 2009; Garyfallidis et al., 2012; Yeatman et al., 2012). Candidate false positive streamlines were excluded from this selection of the most robust streamline. Diffusion metrics included fractional anisotropy and radial, axial, and mean diffusivity by fitting the diffusion tensor model (Basser et al., 1994; Kingsley, 2006) in the lowest gradient DWI (b = 2500) dataset with weighted least squares using dtifit in FSL 5.0.9 (Behrens et al., 2003). Two additional diffusion metrics were extracted from the GQI model: GFA and NQA0 (Yeh et al., 2010).

#### 2.2.6 Manual tracing of the fornix

To establish an anatomically valid target for evaluation of the diffusion reconstruction algorithm and quantitative diffusion metrics, manual tracings of the fornix bundle were performed on the T1-weighted anatomical images by a single rater (TH) blinded to all clinical characteristics. The protocol was similar to that described by Brown et al. (2017), modified as informed by protocols described by Copenhaver et al. (2006) and Amaral et al. (2016). Tracing was performed in FSLView (University of Oxford, England) using a magnified field of view. Intensity was scaled from 0 to 120% of the maximum value and the view was centered on the midline of the anterior commissure. Sagittally, the field of view was magnified to extend from the dorsal aspect of the corpus callosum to the midline of the pons and from the rostral aspect of the genu to the caudal aspect of the splenium. Coronally, the magnified field of view extended from the dorsal aspect of the lateral ventricle to the midline of the pons and from the left-most to the right-most aspect of the inferior horns of the lateral ventricle. Axially, the magnified field of view extended from the rostral to the caudal aspects of the lateral ventricles and from the pial surface on the left to the right. The fornix was traced on consecutive sagittal slices beginning at the midline and moving laterally to the left, then to the right. The coronal and axial views were used to verify tracings and to identify stopping points. Co-registered T2-weighted images were used as reference when needed to verify contrast differences (as between gray and white boundaries). The manual tracings included the body and the crus of the fornix, and the posterior aspect of the fimbria. Tracing of the body was stopped ventrally at the point corresponding to the first axial slice on which the columns of the fornix were clearly separated. Tracing of the fimbria stopped at the sagittal slice in which the hippocampal sulcus was no longer visible. The columns of the fornix, the alveus, and the anterior extent of the fimbria were not traced because they would not be included in the algorithmic method. Although manual tracing has an inherently subjective component, conservative criteria for labeling each voxel were applied to limit partial volume contamination. Volume of each manually traced mask was computed. Diffusion-weighted images were co-registered to the T1 image space for each participant. Diffusion metrics of fractional anisotropy, radial, axial, and mean diffusivity, GFA, and NQA0 were extracted from each manually traced mask and averaged across all voxels in the mask.

#### 2.2.7 Streamline tractography for the genu of the corpus callosum

We applied a similar tractography methodology to the genu of the corpus callosum in each participant to determine whether diffusion characteristics were different between groups in another major bundle with curved anatomy that was not expected to be altered in Alzheimer's disease patients (Head et al., 2004; Di Paola et al., 2010). After manually identifying the most anterior coronal slice where the SDF image displayed a continuous connection of the corpus callosum (Supplementary Fig. S1-A), we moved 3 slices anteriorly and manually created two square ROIs (5 × 5 voxels) centered on the most anterior-posterior SDFs indicative of the anatomy of the genu on the left and right side (Supplementary Fig. S1-B). We performed streamline tractography using the two ROIs as filtered-in regions, applying 10,000 seeds in the left ROI, a 40-degree turning angle, a minimum and maximum length of 40 and 110, and using the automatically-calculated QA threshold using Otsu's method (Otsu, 1975). We trimmed these streamlines to contain only the portion between the ROIs (Supplementary Fig. S1-C, cyan) and isolated the streamline containing the highest average fractional anisotropy (Supplementary Fig. S1-D, white), from which we extracted fractional anisotropy and radial, axial, and mean diffusivity, as well as GFA and NQA0, as above.

### 2.3 Statistical analyses

Statistical analyses were performed using the statistical toolbox in Matlab R2016a (The Mathworks Inc., Natick, MA). Parametric analyses of variance (ANOVA) and chi-square tests were used to evaluate group differences in clinical and demographic characteristics. To investigate structural differences in the fornix or genu bundles, we used general linear models with volumetric and diffusion metrics (e.g. fornix bundle volume, fractional anisotropy, and radial, axial, and mean diffusivity, GFA, and NQA0) as dependent variables, diagnosis (normal control or Alzheimer's disease) as the independent variable, and co-varying for head motion. Estimated fornix volume was used as a covariate in analyses of the diffusion metrics. Fimbriae volumes were also entered as covariates when fimbriae ROIs were used as seeds. Absolute head motion during the scan acquisition was calculated using the b0 images spaced at every thirteenth diffusion image during acquisition. Because the diagnostic groups were age- and sex-matched, age and sex were not included as covariates. Due to our strong directional hypotheses that diffusion measures would be impaired in Alzheimer's disease relative to controls, alpha was set to 0.05 one-tailed for all analyses. Regional analyses of voxels along the fornix bundle were performed using ‘randomise’, a non-parametric permutation-based tool that corrects for multiple comparisons (Nichols and Holmes, 2002) and includes threshold-free cluster enhancement (Smith and Nichols, 2009). We set permutations to 5000 and described significant voxel locations at p ≤ 0.05 corrected.

## 3 Results

### 3.1 Group characterization

Table 1 shows demographic characteristics, head motion during diffusion MRI acquisition, and MMSE scores of Alzheimer's and control participants. Due to matching, there were no significant differences in age or sex between the groups. There was no significant difference in head motion between groups (p = 0.053); nonetheless, head motion was used as a covariate in all subsequent analyses due to its potential impact on data quality (Mukherjee et al., 2008; Yendiki et al., 2014). As expected, Alzheimer's disease participants performed significantly worse on the MMSE.

### 3.2 Reconstruction and volumetric analyses

The volume estimates derived from the tractography reconstruction are displayed by group in Supplementary Table S1 and Supplementary Fig. S2. The estimated fornix volume did not differ significantly between the Alzheimer's disease and normal control groups in the left or right hemisphere either before (p_Left_ = 0.36, p_Right_ = 0.11) or after trimming the streamlines based on the ROI anatomical landmarks (p_Left_ = 0.49, p_Right_ = 0.08, Table 2).

**Table 2:** Statistical results comparing Alzheimer's disease and normal control groups for reconstruction of a continuous fornix bundle using streamline tractography. Beta values indicate the linear effect for normal controls relative to Alzheimer's disease. n = number of observations; SE = standard error; *p < 0.05 (one-tailed).

		Tractography-derived estimated fornix volume	
		Untrimmed streamlines	Trimmed streamlines
Left	β	21.89	0.54
n = 40	SE	68.77	22.05
	p-Value	0.36	0.49
Right	β	85.23	32.32
n = 46	SE	68.51	22.15
	p-Value	0.11	0.08

### 3.3 Diffusion analyses of the fornix bundle

Quantitative differences from diffusion metrics extracted from the most robust streamline are displayed by group in Supplementary Table S1 and Supplementary Figs. S3 and S4. Controlling for fornix volume, (whether using the trimmed estimated volume, Table 3, or the untrimmed estimated volume, not shown) Alzheimer's disease participants had significantly lower fractional anisotropy, and higher radial and mean diffusivity in both the left and right hemispheres (all p ≤ 0.025). No significant group differences were observed for axial diffusivity in either hemisphere (all p ≥ 0.30). Metrics from the generalized sampling imaging reconstruction showed that Alzheimer's disease participants had significantly lower GFA and NQA0 (all p ≤ 0.003). All results were unchanged when fornix volume was removed as a covariate. Relationships between the trimmed volume estimates and each diffusion metric are displayed in Supplementary Figs. S5 and S6. These results indicate that multiple diffusion metrics detect differences between the two diagnostic groups even when total fornix volume is comparable. Examining the genu of the corpus callosum as a control tract, Alzheimer's participants showed significantly lower NQA0 (p = 0.035) while all other diffusion metrics showed no significant differences (all p ≥ 0.22, Supplementary Table S2).

**Table 3:** Statistical comparison between Alzheimer's disease and normal control groups for diffusion tensor metrics from the fornix bundle centerline in the left and right hemisphere, controlling for trimmed fornix volume. Trimmed fornix volume, head motion and fimbria volume were included as covariates in each model. Results include diffusion tensor metrics: fractional anisotropy (FA), radial diffusivity (RD), axial diffusivity (AxD), and mean diffusivity (MD) and generalized Q-imaging metrics: generalized fractional anisotropy (GFA) and normalized principal quantitative anisotropy (NQA0). β denotes the linear effect for normal controls relative to Alzheimer's disease. SE = standard error; *p < 0.05 (one-tailed).

		Diffusion tensor metrics				Generalized Q-imaging metrics	
		FA	RD	AxD	MD	GFA	NQA0
Left	β	0.065	−6.23e−5	91.3e−5	−5.23e−5	0.02	0.055
n = 40	SE	0.021	2.8e−5	2.0e−5	2.41e−5	0.007	0.015
	p-Value	0.0023*	0.015*	0.32	0.018*	0.002*	<0.001*
Right	β	0.084	−9.74e−5	−78.9e−5	−6.76e−5	0.025	0.07
n = 46	SE	0.019	2.64e−5	2.16e−5	2.23e−5	0.006	0.014
	p-Value	<0.001*	<0.001*	0.36	0.002*	<0.001*	<0.001*

### 3.4 Diffusion analysis of the fornix bundle along its trajectory

**Fig. 4:**
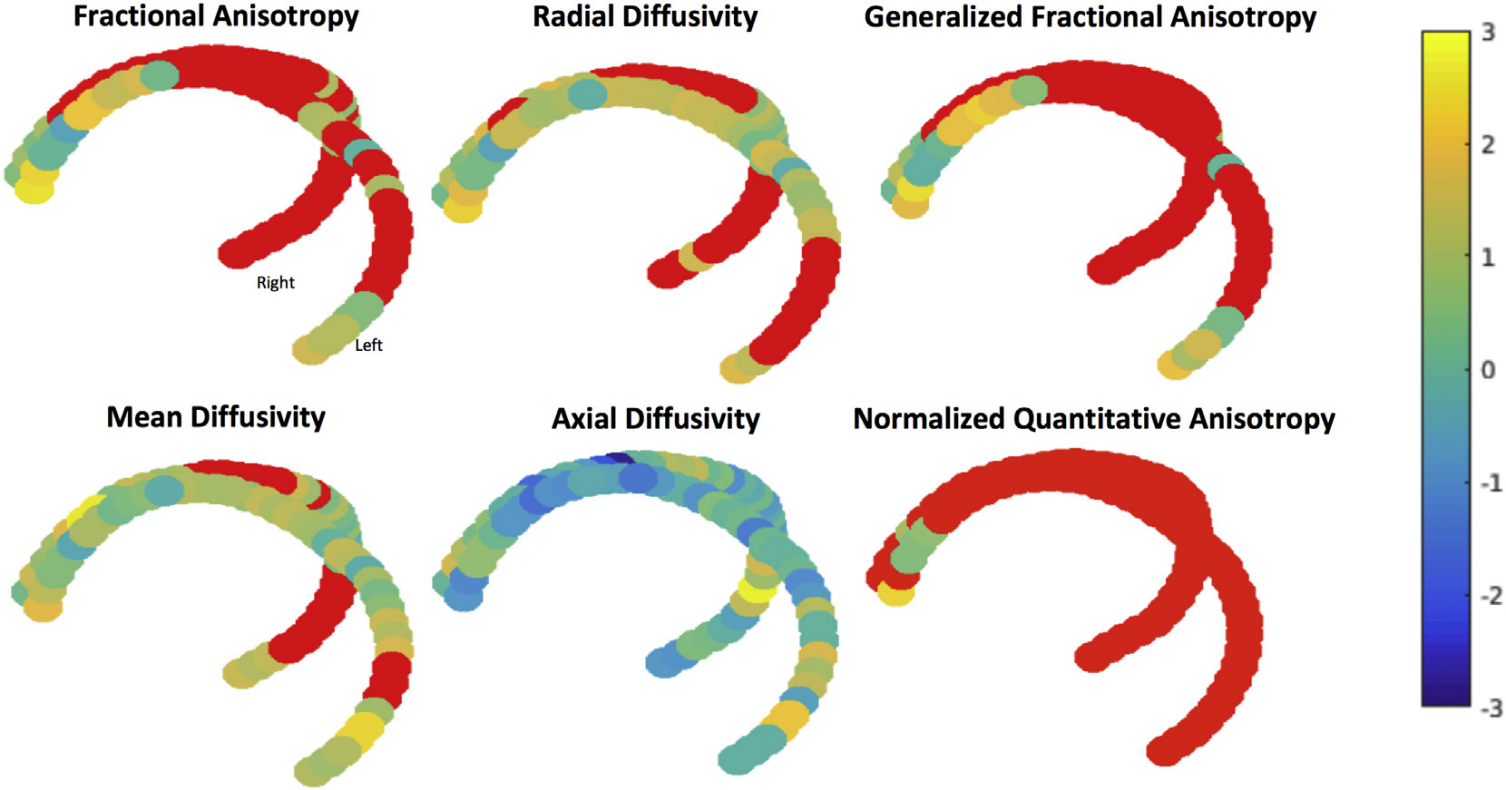
Voxel-by-voxel values comparing Alzheimer's disease with normal controls in the four diffusion metrics of interest. Colors indicate t-values, and are scaled such that normal controls exhibit higher fractional anisotropy and lower radial, axial, and mean diffusivity than the Alzheimer's disease group. Red points indicate threshold-free cluster enhancement corrected p < 0.05 using a non-parametric permutation test.

When examining the voxel-by-voxel estimates of each diffusion metric along the most robust streamline fornix bundle, many voxels had significantly lower fractional, GFA, NQA0 and higher radial diffusivity in Alzheimer's disease relative to normal controls, with the crus and body of the fornix most affected (Fig. 4). Fewer voxels had significantly higher mean diffusivity in Alzheimer's disease relative to normal controls, but these were also localized to the crus and body. The overall pattern of altered diffusion across multiple voxels indicates that the observed effects are not primarily localized to a single region or hemisphere. There were no significant voxels for axial diffusivity. Notably, there were no voxels along the most robust streamline where any metric demonstrated significantly more intact diffusion in Alzheimer's disease than in normal controls.

### 3.5 Manual tracing analyses

To verify that the results we observed using the algorithmic identification of the fornix possessed anatomic validity, we compared results with those from a manual tracing method. The volume of the manual tracings did not differ significantly between the Alzheimer's disease and normal control groups (left p = 0.26, right p = 0.31, Table 4). Diffusion metrics extracted from the voxels identified by manual tracings found significant differences by diagnosis for fractional anisotropy, radial diffusivity, mean diffusivity, GFA, and NQA0 (all p ≤ 0.005), but not for axial diffusivity (left p = 0.26, right p = 0.11), controlling for motion during the diffusion scans and for volume from the manual tracing (Table 4). If volume is not included as a covariate, the results are unchanged. The results from the manual tracings (Table 4) were highly convergent with those from the algorithmic method (Table 3). The correlations between diffusion metrics extracted from the manual tracing method and the algorithmic method (averaging the left and right) were r = 0.70 for fractional anisotropy, r = 0.85 for radial diffusivity, r = 0.60 for axial diffusivity, r = 0.86 for mean diffusivity, r = 0.67 for GFA, and r = 0.81 for NQA0.

**Table 4:** Statistical comparison between Alzheimer's disease and normal control groups for volume and diffusion metrics of the fornix bundle derived from manually traced segmentations. Diffusion metrics were transformed into T1 space and linear models were controlled for diffusion motion and T1 fornix volume. Diffusion tensor metrics include fractional anisotropy (FA), radial diffusivity (RD), axial diffusivity (AxD), and mean diffusivity (MD). Generalized q-imaging metrics include generalized fractional anisotropy (GFA) and normalized principal quantitative anisotropy (NQA0). β denotes the linear effect for normal controls relative to Alzheimer's disease. SE = standard error, *p < 0.05 (one-tailed).

		T1	Diffusion tensor				Generalized sampling imaging	
		Fornix volume	FA	RD	AxD	MD	GFA	NQA0
Left	β	23.26	0.05	−7.4E−5	−1.4E−5	−5.4E−5	0.016	0.041
n = 46	SE	36.03	0.019	2.38E−5	2.17E−5	1.97E−5	0.006	0.013
	p-Value	0.26	0.004*	0.0017*	0.26	0.005*	0.005*	0.002*
Right	β	−0.30	0.07	−10.2E−5	−2.6E−5	−7.6E−5	0.02	0.056
n = 46	SE	36.72	0.019	2.13E−5	2.13E−5	1.75E−5	0.006	0.014
	p-Value	0.99	<0.001*	<0.001*	0.11	<0.001*	<0.001*	<0.001*

## 4 Discussion

Our primary aim was to determine whether and where the microstructure of the fornix bundle is significantly degraded during Alzheimer's disease relative to age- and sex-matched normal older adults using enhanced diffusion measurement techniques, when controlling for potential volumetric differences. To accomplish this, we reconstructed a continuous fornix bundle using high gradient diffusion-weighted images collected from the MGH-USC CONNECTOM MRI scanner and applied streamline tractography with robust streamline isolation techniques to compare volume and diffusion metrics of the fornix bundle in a sample of Alzheimer's disease versus age- and sex-matched normal controls. In contrast to previous findings (Copenhaver et al., 2006; Amaral et al., 2016), we did not observe significant fornix volume differences between Alzheimer's disease patients and normal controls. Consistent with previous findings (Bozoki et al., 2012; Oishi et al., 2012; Fletcher et al., 2013; Kantarci, 2014; Amaral et al., 2016), the Alzheimer's disease group had more impaired values compared to normal controls in diffusion metrics derived from the tensor model (fractional anisotropy, radial diffusivity, and mean diffusivity) and the generalized q-imaging model (GFA and NQA0). These results corresponded well to results from manual tracings of the fornix. Analysis along the fornix trajectory suggested that the left and right crus and body of the fornix were the most affected regions. Similar analyses for the genu of the corpus callosum found no significant group differences except for NQA0, suggesting that there is at least some specificity to the fornix (see Supplementary material). The genu was chosen as a control tract based on its curved anatomy and evidence that it may not be as impacted in Alzheimer's disease in age-matched samples (Head et al., 2004; Di Paola et al., 2010).

While the fornix bundle has been a target of high interest in Alzheimer's disease (Ringman et al., 2007; Huang et al., 2012; Fletcher et al., 2013; Jin et al., 2015) and its diffusion characteristics may provide a biomarker for progression along the Alzheimer's disease spectrum (Oishi et al., 2012; Nowrangi and Rosenberg, 2015), its utility as such a biomarker has been limited by challenges to accurate measurement with diffusion tractography. These challenges result from its intrinsic morphology, including its highly curved anatomy and its proximity to CSF that diminishes diffusion signal. The problems these characteristics pose for accurate tractography become especially evident in the crus of the fornix where diverging fibers form the hippocampal commissure (Daitz, 1953), and increase when atrophy leads to larger partial volume effects, as occurs in older populations (Salat, 2011) and Alzheimer's disease patients (Sexton et al., 2011; Acosta-Cabronero and Nestor, 2014). Further, the reconstruction of a continuous fornix bundle is more difficult when few diffusion gradient encoding directions (≤30) are used (Concha et al., 2005; Ringman et al., 2007; Fletcher et al., 2013), and when partial volume effects from adjacent CSF contamination (Metzler-Baddeley et al., 2012) may be introduced by low spatial resolution or normalization to an atlas-derived template (Oishi et al., 2012; van Bruggen et al., 2012; Zhuang et al., 2013; Oishi and Lyketsos, 2014). Some prior approaches have mitigated some of these challenges by manually extracting diffusion values in the columns or/and body of the fornix (Ringman et al., 2007; Mielke et al., 2009; Nowrangi et al., 2013; Boespflug et al., 2014), which is least likely to be contaminated by CSF, but at the cost of providing an incomplete picture of fornix diffusion characteristics.

In the present work, we attempted to address the above challenges by 1) acquiring multi-shell high-gradient diffusion-weighted images from a first-of-its-kind MRI scanner to enable increased signal-to-noise ratio and to resolve complex white matter tracts (Fan et al., 2014), 2) acquiring many diffusion encoding gradient directions (268 directions compared to the conventional ~30–60 directions) to enable estimation of a multi-fiber population diffusion model, and 3) applying an optimized streamline tractography and streamline isolation protocol in each participant's native space to reconstruct a continuous fornix bundle and extract voxel-by-voxel estimates of diffusion metrics along its trajectory.

Our primary finding was that fractional anisotropy, radial diffusivity, mean diffusivity, generalized fractional anisotropy and normalized quantitative principal anisotropy were significantly worse in Alzheimer's disease patients relative to their age- and sex-matched normal control counterparts even when controlling for the estimated volume of the fornix. In all analyses, we controlled for head motion, and where appropriate, for the size of the fimbria ROIs. These results were highly consistent with those derived from manual tracings. These results suggest that diffusion characteristics that measure changes in the directional diffusion of water along myelinated axons of the fornix are sensitive to Alzheimer's disease-related neurodegenerative processes. These findings are largely consistent with previous comparisons of diffusion characteristics in the fornix in Alzheimer's disease, despite the fact that prior comparisons have typically investigated only the body (Mielke et al., 2009; Huang et al., 2012; Nowrangi et al., 2013) or columns (Ringman et al., 2007) of the fornix, have conducted analyses in normalized space (Huang et al., 2012; Metzler-Baddeley et al., 2012; van Bruggen et al., 2012; Berlot et al., 2014), or have used atlas-based measurements (Pievani et al., 2010; Jin et al., 2015; Brown et al., 2017). Our continuous bundle analyses extend these previous findings to demonstrate that Alzheimer's disease impacts diffusion characteristics throughout the fornix bundle. Our method is advantageous because it results in a complete reconstruction between consistent landmarks along the fornix bundle to allow extraction of diffusion characteristics from comparable anatomy in each participant.

One point of inconsistency with some prior studies is our finding of no significant differences between Alzheimer's disease and normal control participants for axial diffusivity along the fornix bundle. Although not always present (e.g., Huang et al., 2012), several studies have reported significantly *higher* fornix axial diffusivity in Alzheimer's disease patients than in controls (Acosta-Cabronero et al., 2010; Pievani et al., 2010; Ryan et al., 2013; Acosta-Cabronero and Nestor, 2014). These reports are somewhat incongruous with expectations, as axial diffusivity measures the rate of diffusion along the principal axis of the fiber bundle. Although the fornix bundle has a highly curved anatomy, it has a primarily anterior-to-posterior direction with a predominantly uniform directionality at any given point. There are three major routes by which fornix degeneration in Alzheimer's disease would be expected to affect diffusion values (Badea et al., 2016). First, fiber degeneration would result in a lower density of fibers per voxel. Second, degradation of myelin sheaths would result in greater diffusion in directions perpendicular to the axon (hence higher radial diffusivity, see Song et al., 2002; Budde et al., 2007). In these cases, one would expect the primary axis of diffusion to be lower in Alzheimer's disease patients as diffusion of water moves from the principal axis to other directions. One would therefore expect higher radial and mean diffusivity, but similar or lower axial diffusivity in the fornix for Alzheimer's disease patients. Third, overall volume loss would result in partial volume effects from surrounding CSF. In this case, higher axial diffusivity may be expected to the extent that the absence of tissue constraints in CSF results in water diffusing more quickly (Alexander et al., 2007). Higher axial diffusivity may therefore be an indication of likely partial volume effects, although alternative explanations have been formulated. In other fiber tracts, findings of higher axial diffusivity in Alzheimer's disease have been postulated to reflect a reduction of crossing fibers that results in a higher principal axis of diffusion because fibers running in other directions are no longer present (Douaud et al., 2011; Teipel et al., 2014). This potential explanation encounters difficulty in that fewer crossing fibers would also result in lower radial diffusivity and higher fractional anisotropy values in the patients, so that multiple factors must be invoked to account for different diffusion metrics. This explanation is also unlikely to apply to the fornix, which is largely free from crossing fibers (Acosta-Cabronero et al., 2012; Acosta-Cabronero and Nestor, 2014). Although the crus possess diverging fibers that form the hippocampal commissure, within a voxel in close proximity to the crus these fibers likely run mostly parallel to the principal axis. Of note, when using diffusion metrics from the GQI model, we found a significant decrease for NQA0 in the Alzheimer's disease sample. While axial diffusivity and NQA0 both describe the principal axis of diffusion, they measure different biophysical properties along this axis. Axial diffusivity describes the rate of diffusion, whereas NQA0 describes the density of diffusion spins along the principal axis (Yeh et al., 2013). Our findings suggest that NQA0 may be more sensitive to slight degradation of the myelin sheath even in the absence of volumetric declines, whereas axial diffusivity may require a greater extent of damage before differences can be observed.

While our study was primarily focused on diffusion characteristics of the fornix, our methods also provide individualized estimates of fornix volume. Our results found no significant differences between Alzheimer's disease and normal control participants using our estimates of fornix volume from tractography or using manually traced volumes. We note that this is inconsistent with previous fornix volume findings using manual tracing or semi-automatic atlas methods (Copenhaver et al., 2006; Amaral et al., 2016). One possibility is that our sample size was insufficient to detect volume effects. As seen in Supplementary Table S1, the estimated fornix volumes were lower (albeit not significantly) in the Alzheimer's disease group than in the normal control group, though the magnitude of this difference ranged from a 1.8% to a 14.2% mean difference. Our sample size provided adequate power to detect the larger effect sizes previously reported in studies comparing fornix volume differences between Alzheimer's disease patients and controls (Copenhaver et al., 2006; Amaral et al., 2016); if the true effect sizes are smaller than in these previous reports, studies with larger sample sizes will be necessary. Another possibility is that the lenient threshold in our tractography method induces false positive streamlines that inflate the volume estimates. Manual tracings of individual participants revealed that a continuous fornix bundle extending from the anterior commissure to the tail of the hippocampus was observable in all participants, suggesting that our continuous reconstruction is consistent with the underlying anatomy. For this to be a major contributing factor, such inflation would have to be more likely in the Alzheimer's group than in the control group. Our estimates (Section 2.2.4) suggest that candidate false positives were observed in fewer Alzheimer's disease patients than in the matched controls, with similar average numbers of candidate false positives across groups. We note that our reconstruction method likely underestimates the volume specifically in the body and in the fimbriae because of its requirement that each streamline fully extend between these regions; this may be one source of the inconsistency with other volumetric methods to the extent that those methods are not subject to this limitation. The inconsistency between our manually traced estimates and other manually tracing studies may be due to our exclusion of the columns, the alveus, and the anterior aspect of the fimbria, to differences in patient definition, or to our method of age and sex matching controls and patients. We note that our method provides a conservative estimate of volume differences between diagnostic groups in exchange for higher confidence that the diffusion values are drawn from voxels that represent true fornix bundle anatomy.

Although our techniques for measuring diffusion characteristics of the fornix have several favorable aspects, it is important to note multiple caveats and limitations (Jones et al., 2013). First, while our techniques likely minimize partial volume effects by remaining in native diffusion space and extracting values from the most robust streamline least likely to be contaminated by CSF, partial volume effects will still occur to some extent owing to the resolution of our diffusion acquisition (Vos et al., 2016). The body of the human fornix (subcallosal anterior to psalterium) has been estimated as having a cross-sectional area of ~25 mm^2^, with approximately 2,700,000 fibers of 1 μm in diameter on the left and right sides (Daitz, 1953). This means that in-plane images of the fornix body will be subsumed within a 3 × 3 voxel grid at current resolution. The anterior pillars were estimated to have a cross-sectional area of only 3 mm^2^ (Daitz, 1953). In our manual tracings of individual participants, the crura of the fornix were observed to be limited to a single 1 mm^3^ voxel in the volumetric T1 images in multiple individuals. These findings indicate that the anterior pillars and the crus would be fully subsumed within a single 1.8 mm^3^ diffusion voxel in our data. This implies that partial volume effects likely occur even within a single diffusion voxel in some regions of the fornix. To the extent that Alzheimer's disease participants are more likely to be susceptible to such partial volume effects than are normal control participants, differences in the diffusion metrics will be overestimated. Against this concern, our method of extracting the diffusion metrics from the most robust streamline for each individual requires that each participant will be evaluated using a streamline that is highly likely to exhibit intact values, which should produce a conservative estimate of differences between Alzheimer's disease and normal control participants in the diffusion metrics. Second, our methods are semi-automated in nature, requiring some manual interaction for placement of the spherical ROIs within the body of fornix. This small degree of manual interaction may be considered a drawback for use with larger datasets, although it is substantially less labor-intensive than fully manual tracing methods. Third, although no co-registration was used on the diffusion imaging data to be analyzed, we did use co-registration tools to warp the most canonical ROA into every participant's native diffusion space. Although this could introduce error, it is unlikely because the ROA is large enough to fully subsume the structure of the fornix and the fimbria in all our participants. Fourth, the method uses automatically parcellated fimbriae ROIs from the Freesurfer software (Wisse et al., 2017) and will be limited by the accuracy of this parcellation scheme. These methods will likely see improvement in future versions of Freesurfer. When especially small fimbriae parcellations were encountered, we dilated these (limiting the dilation to voxels in the hippocampal segmentation) to capture bundles that may nonetheless belong to the fornix. If this method were to introduce spurious streamlines not part of the morphology of the fornix, these should be filtered out by each participant's ROA. To account for potential differences related to the fimbriae reconstructions, we corrected for fimbria volume in our models when appropriate. Fifth, because our method of tractography is optimized to fibers in the anterior-to-posterior direction, we do not measure the hippocampal commissure, which crosses the cerebral hemispheres. Our method also excludes the columns of the fornix and the alveus from the most robust streamline used to quantitate across participant statistics. This is a consequence of using standardized landmarks to increase the likelihood that diffusion metrics are extracted from comparable anatomy for each participant.

An additional caveat is that we did not acquire verification of amyloid status for most of our participants. Through ancillary studies, amyloid imaging using Pittsburgh Compound B was available for 21 participants (5 Alzheimer's disease patients and 16 controls). All 5 Alzheimer's disease patients were classified as amyloid positive, while all 16 controls were classified as amyloid negative (see methods). Of the remaining 7 control participants, approximately 30%, or 2 participants, should be expected to present as amyloid positive were imaging available (e.g., Chetelat et al., 2013). For the 5 Alzheimer's disease patients, amyloid imaging data were examined after enrollment and were not used to exclude amyloid negative patients – we simply did not encounter any such cases. Because these data were not acquired on all participants, these findings cannot be considered conclusive; however, they are suggestive that the diagnostic labels correspond to amyloid status of the participants. Nonetheless, it is important to note that approximately 15% of diagnosed Alzheimer's disease patients present as amyloid negative at autopsy (Ossenkoppele et al., 2015) hence, disruption of diffusion characteristics of the fornix in clinically identified Alzheimer's disease may not be directly associated with amyloid accumulation.

Our findings of alterations in diffusion characteristics of the fornix in the absence of volumetric differences add support for models of Alzheimer's disease as a disconnection disorder (Morrison et al., 1986; Delbeuck et al., 2003; Bartzokis et al., 2004; Catani and ffytche, 2005; Wisse et al., 2015) and provide additional evidence for white matter deterioration in Alzheimer's disease (Brun and Englund, 1986; Brickman et al., 2009; Bartzokis, 2011; Bozoki et al., 2012; Amlien and Fjell, 2014; Lee et al., 2016; Lindemer et al., 2017). While functional connectivity studies (Buckner et al., 2009; Chhatwal and Sperling, 2012; Brier et al., 2014; Schultz et al., 2017) also support disconnection models, structural connectivity via white matter has been less well investigated in Alzheimer's disease (Matthews et al., 2013; Daianu et al., 2015). Despite the present study's focus on the fornix as a potential contributor to disconnection of the hippocampus, other white matter pathways are likely important for understanding how Alzheimer's disease pathology leads to cognitive decline.

## 5 Conclusion

The primary aim of this study was to determine whether diffusion characteristics in the fornix bundle can be measured independently of volumetric decreases of the fornix that may occur with Alzheimer's disease. Comparing Alzheimer's disease patients with age- and sex-matched normal controls and using hardware enhanced streamline tractography protocols to reconstruct a continuous fornix, significant alterations were observed in the Alzheimer's disease group compared to the normal controls in multiple diffusion metrics despite no significant differences in volume measurements. These results suggest that diffusion characteristics may provide sensitive measures of fornix degeneration related to Alzheimer's disease over and above volumetric measures of the fornix. Of note, our novel fornix reconstruction method resulted in reconstruction of complete fornix bundles bilaterally in most participants, even in the face of readily apparent morphometric alterations in some participants. Because our diffusion metrics were taken from the fornix streamline with the highest average fractional anisotropy in each participant, the observed differences provide a conservative estimate of the impact of Alzheimer's disease on fornix microstructure. Although based on cross-sectional and group-level findings, these results support an interpretation in which microstructural degeneration of the fornix bundle is followed by macrostructural volume loss of the fornix in Alzheimer's disease. Diffusion characteristics of the fornix may therefore provide a useful and complementary biomarker target during the progression of Alzheimer's disease. While the clinical utility of these methods is currently limited by the necessity of collecting many diffusion directions, the accompanying long acquisition time, and the limited availability of the high gradient scanner, the biology of the fornix uncovered by this study suggests that diffusion characteristics may provide a sensitive marker of the impact of neurodegeneration on connectivity of the hippocampus. With greater availability of high gradient scanners and advancements allowing compression of acquisition time (Setsompop et al., 2013; Fan et al., 2014; Fan et al., 2016), diffusion imaging studies using increased sample sizes, additional diagnostic groups (e.g., mild cognitive impairment), and focused on other white matter pathways will be feasible to increase our understanding of the contribution of impairments in structural connectivity during the progression of Alzheimer's disease.

## Funding

Funding was provided by the National Institutes of Health, National Institute on Aging [grants P50 AG005134, R01 AG053509, P01 AG036694, and K01 AG040197], and National Institute of Mental Health [grant U01 MH093765]. JSR was supported by an award from the Canadian Institutes of Health Research. This research was carried out in part at the Athinoula A. Martinos Center for Biomedical Imaging at the Massachusetts General Hospital, using resources provided by the Center for Functional Neuroimaging Technologies, P41EB015896, a P41 Biotechnology Resource Grant supported by the National Institute of Biomedical Imaging and Bioengineering (NIBIB), National Institutes of Health. This work also involved the use of instrumentation supported by the NIH Shared Instrumentation Grant Program and/or High-End Instrumentation Grant Program [grants S10RR023401, S10RR019307, S10RR019254, and S10RR023043].

## Appendix A Supplementary data

Supplementary Tables and Figures. Image 1

Fornix ROI and ROA templates. Image 2
