# Supplementary material for: Connectome-derived diffusion characteristics of the fornix in Alzheimer's disease
Source: Neuroimage Clin. 2018 Apr 27;19:331–42. doi: 10.1016/j.nicl.2018.04.029 (PMC6044183; doi:10.1016/j.nicl.2018.04.029)
Supplement: Supplementary file 1 — Supplementary Tables and Figures. [file mmc1.pdf]

	Alzheimer’s disease Mean(SD)		Normal controls Mean(SD)	
**Estimated Volume**	Left	Right	Left	Right
Untrimmed	619.9 (215.4)	500.17(210.12)	635.95 (225.7)	582.8 (204.6)
Trimmed	244.2 (69.6)	221.6 (68.7)	249.2 (75.6)	242.7 (65.5)
**Diffusion Metric**				
FA	0.348 (0.05)	0.318 (0.05)	0.414 (0.08)	0.41 (0.06)
RD	6.4e-3 (6.94e-5)	6.75e-3 (7.38e-5)	5.64e-3 (9.29e-5)	5.63e-3(8.51e-5)
AxD	1.1e-3 (6.15e-5)	1.09e-3 (5.85e-5)	1.09 (8.42e-5)	1.08 (7.46e-5)
MD	0.79e-3 (6.05e-5)	0.82 (6.27e-5)	0.74e-3 (7.58e-5)	0.74e-3 (7.31e-5)
GFA	0.12 (0.015)	0.11 (0.016)	0.141 (0.025)	0.139 (0.021)
NQA0	0.17 (0.03)	0.155 (0.032)	0.22 (0.06)	0.216 (0.05)

Table S1. Mean and standard deviation (SD) values for each volumetric and diffusion metric. FA = fractional anisotropy, RD = radial diffusivity, AxD = axial diffusivity, MD = mean diffusivity, GFA = generalized fractional anisotropy, and NQA0 = normalized principal quantitative anisotropy. Fornix volumes did not reach significant differences between groups. All diffusion metrics except axial diffusivity were significantly different between groups.

		Diffusion Tensor Metrics				Generalized Q-Imaging Metrics	
		FA	RD	AxD	MD	GFA	NQA0
Genu of the	**β**	-0.006	-2.20e-5	-1.55e-5	-6.62e-5	0.89e-3	0.025
corpus callosum	SE	0.0097	1.09e-5	1.99e-5	1.23e-5	3.5e-3	0.014
(n=46)	p-value	0.26	0.42	0.22	0.3	0.4	0.035*

Table S2. Statistical comparison between Alzheimer's disease and normal control groups for diffusion metrics from the most robust streamline in the genu of the corpus callosum. Results include fractional anisotropy (FA), radial diffusivity (RD), axial diffusivity (AxD), and mean diffusivity (MD). β denotes the linear effect for normal controls relative to Alzheimer's disease. SE = standard error.

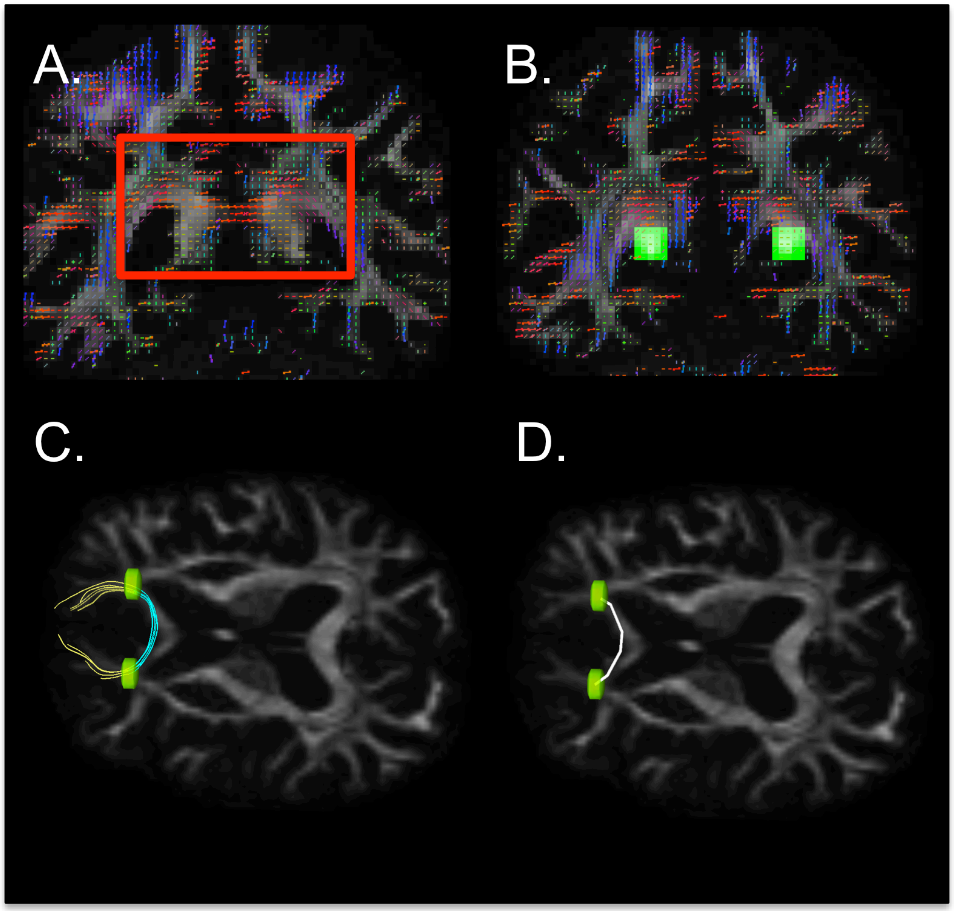

Supplementary Figure S1. Demonstration of selection of the most robust streamline in the genu of the corpus callosum. A) First, we located the most anterior coronal slice where transverse fibers of the genu were visible. B) We then moved three slices anteriorly and placed bilateral ROIs 5x5 voxels in dimension centered on the anterior-posterior spin distribution functions indicative of the anatomy of the genu. C) Using the total number of streamlines that connect these two ROIs, D) we identified the streamline with the highest averaged fractional anisotropy and trimmed the portion extending beyond the defining ROIs.

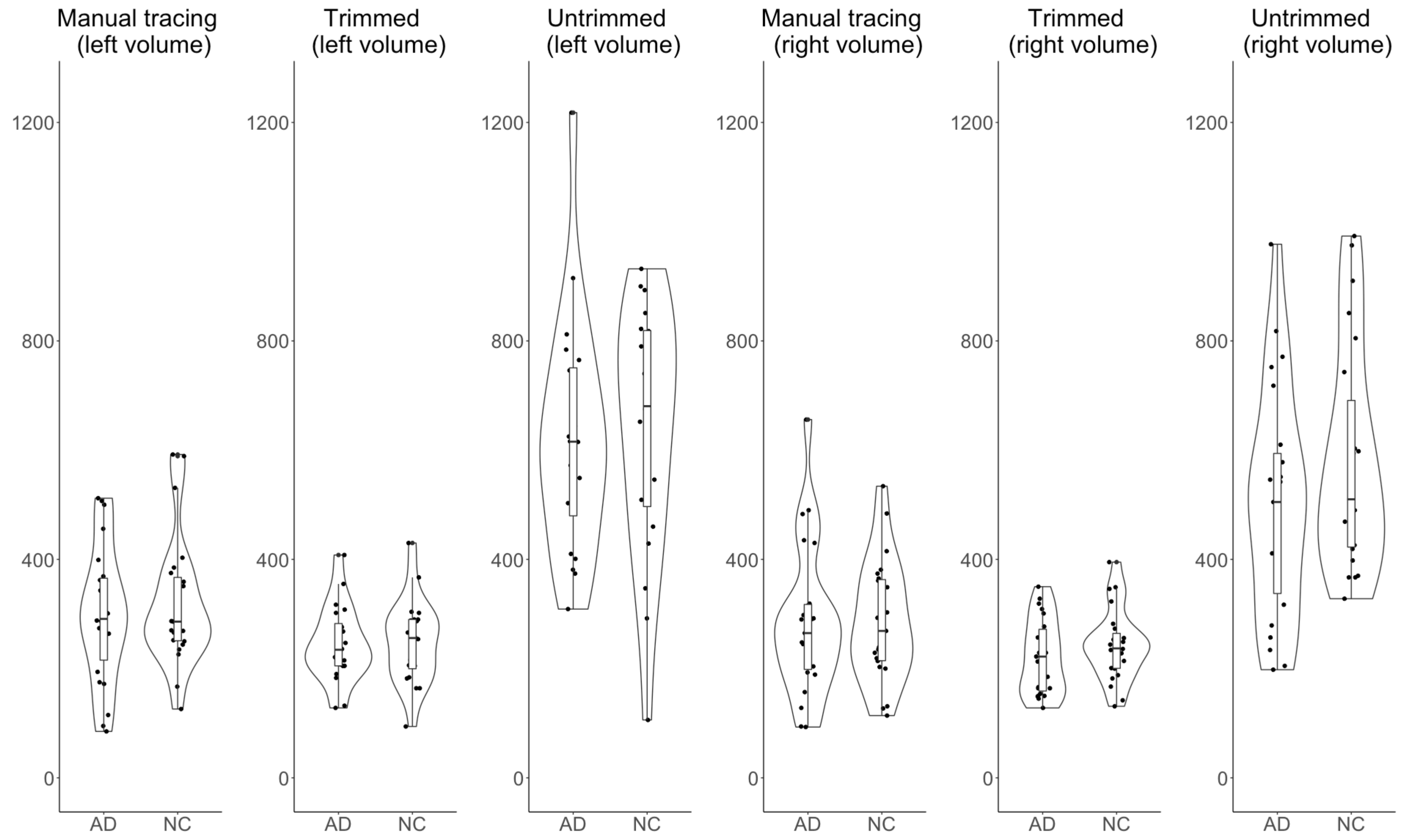

Supplementary Figure S2. Violin plots showing the mean (bar), standard deviation (boxes), 95% confidence intervals (whiskers), and individual values (points) for the Alzheimer's disease (AD) and normal control (NC) groups for each volume estimate, separately for the left and right hemispheres.

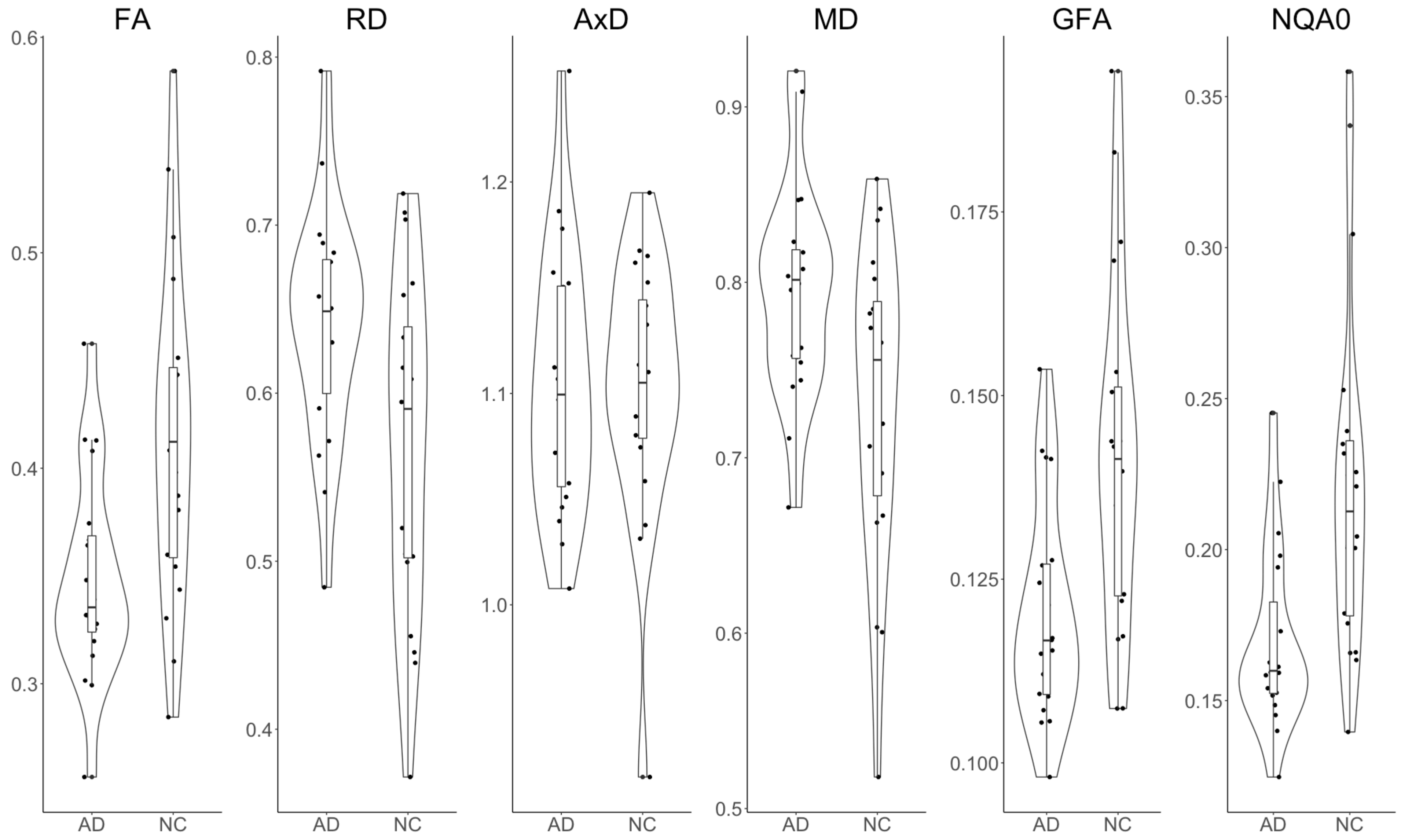

Supplementary Figure S3. Violin plots showing the mean (bar), standard deviation (boxes), 95% confidence intervals (whiskers), and individual values (points) for the Alzheimer's disease (AD) and normal control (NC) groups for each diffusion metric for the left hemisphere.

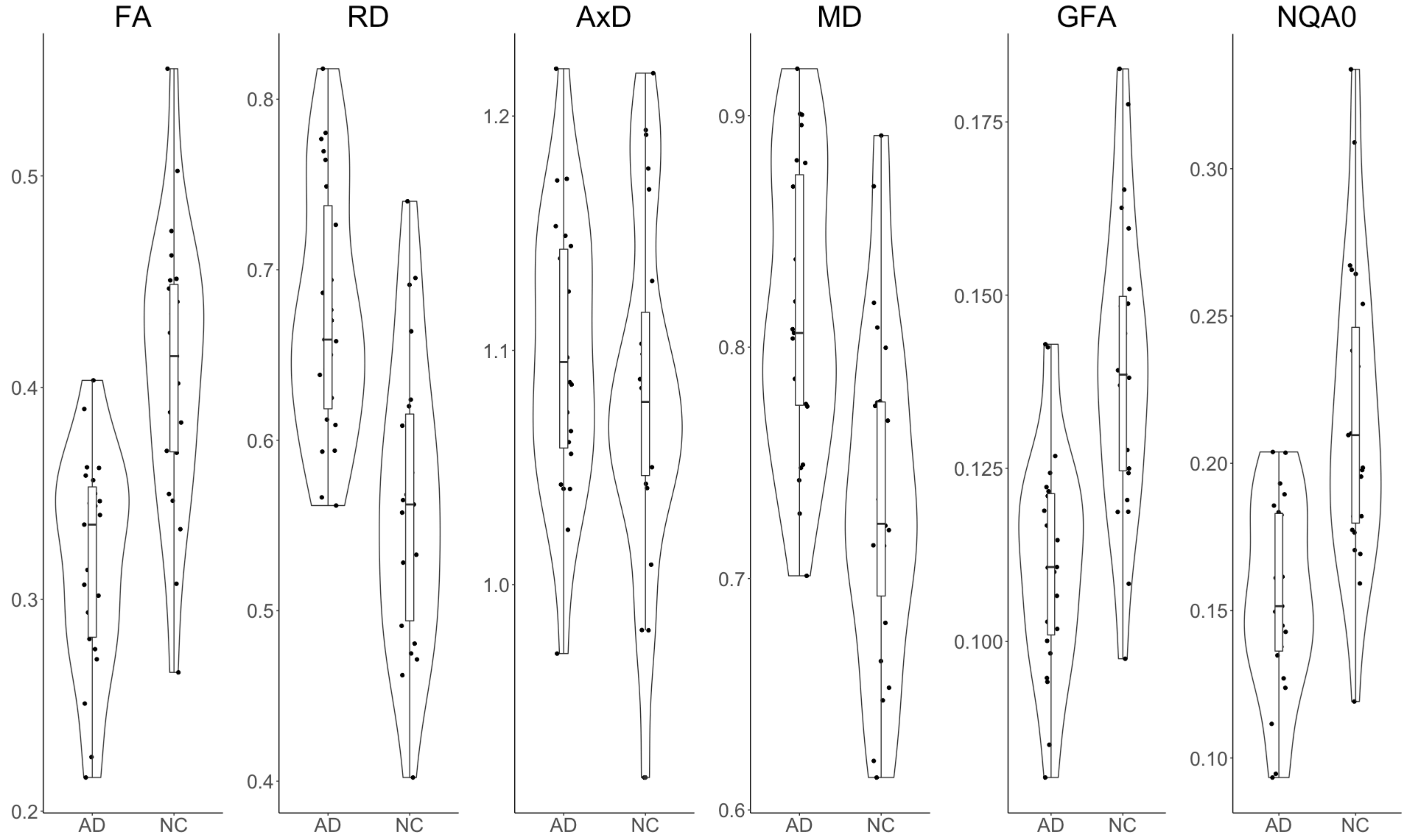

Supplementary Figure S4. Violin plots showing the mean (bar), standard deviation (boxes), 95% confidence intervals (whiskers), and individual values (points) for the Alzheimer's disease (AD) and normal control (NC) groups for each diffusion metric for the right hemisphere.

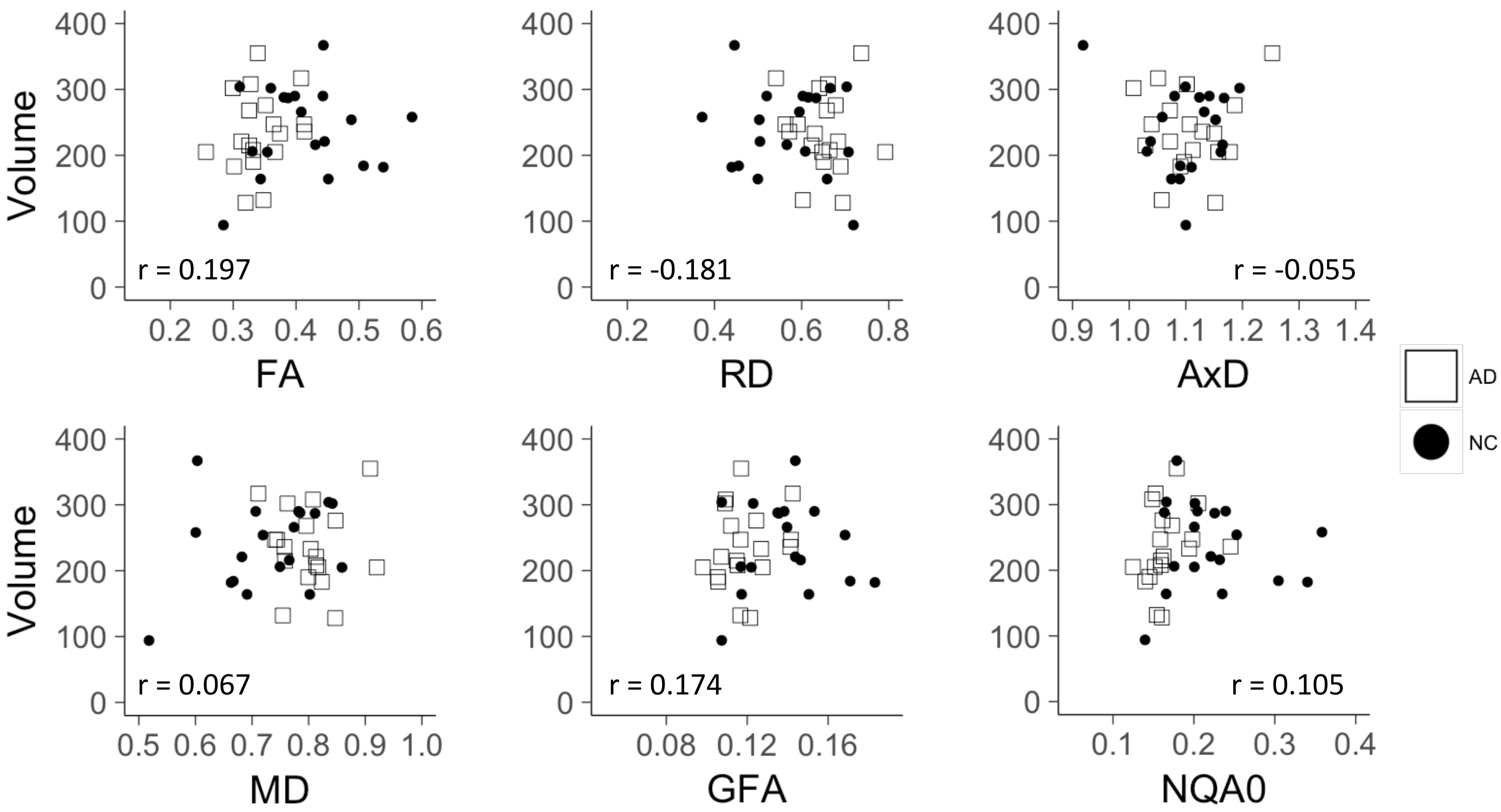

Supplementary Figure S5. Scatterplots showing relationships between the trimmed volume of the left fornix and each diffusion metric (FA, RD, AxD, MD, GFA, and NQA0) for the left hemisphere. The correlation for the total group is displayed on each panel. The correlation was not significantly different between the Alzheimer's disease (AD) and normal control (NC) groups for any diffusion metric.

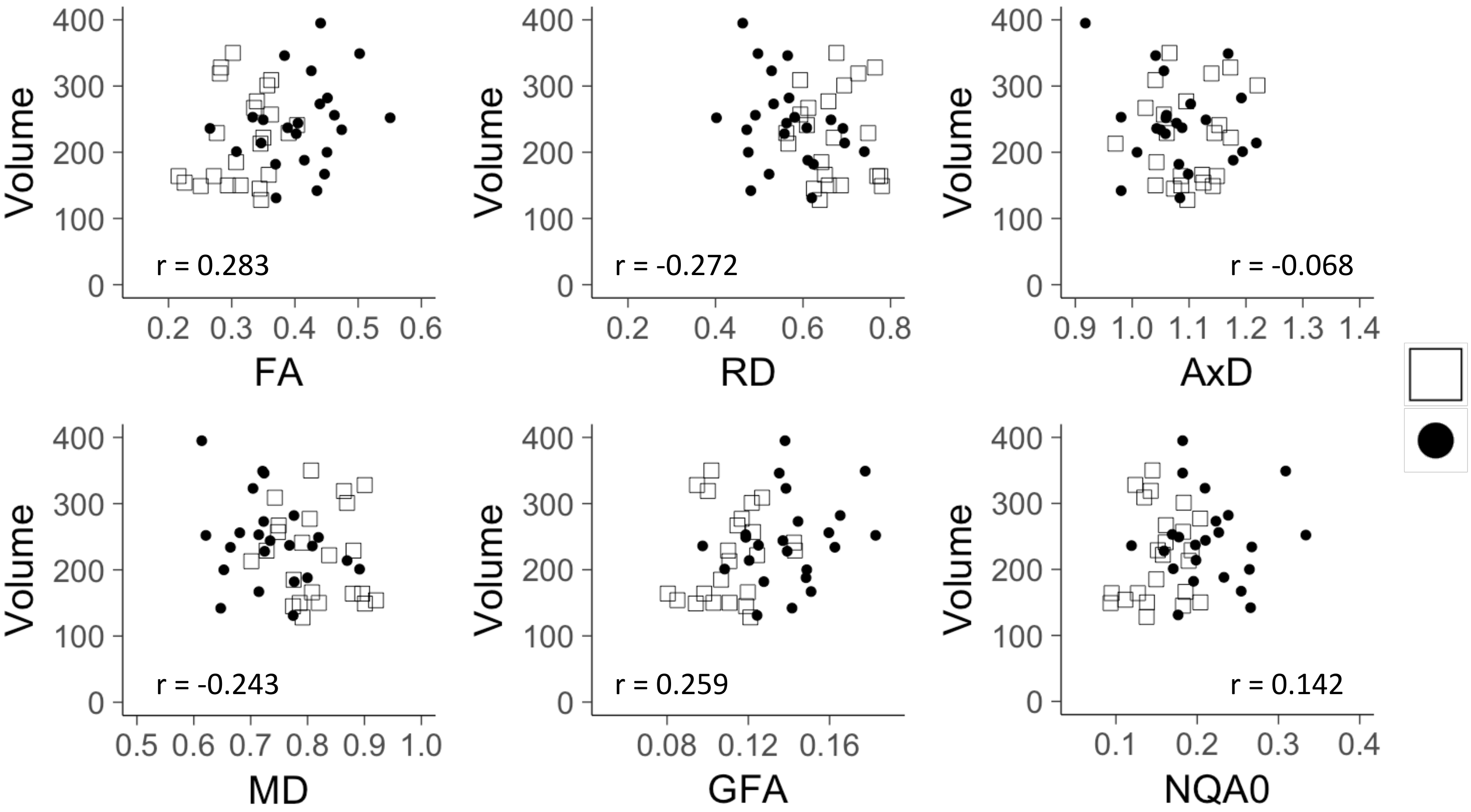

Supplementary Figure S6. Scatterplots showing relationships between the trimmed volume of the right fornix and each diffusion metric (FA, RD, AxD, MD, GFA, and NQA0) for the right hemisphere. The correlation for the total group is displayed on each panel. The correlation was not significantly different between the Alzheimer's disease (AD) and normal control (NC) groups for any diffusion metric.
